# The impact of armed conflict on nursing students’ self-esteem: a cross-sectional comparative study

**DOI:** 10.25122/jml-2024-0063

**Published:** 2024-07

**Authors:** Mohammead Mohammead, Atallah Alenezi, Mohammed Abdelmalik, Fahad Alhowaymel, Mohamed Abdallah, Abdalrahman Saeed, Sara Musa, Elturabi Ebrahim, Shahenda Salih

**Affiliations:** 1 Department of Nursing, College of Applied Medical Sciences, Shaqra University, Shaqra, Saudi Arabia; 2 College of Nursing, Imam Mohammad Ibn Saud Islamic University (IMSIU), Riyadh, Saudi Arabia; 3 Al-Rayan Colleges, Al-Rayan Private College of Health Sciences and Nursing, Al Madinah Al Munawarah, Saudi Arabia; 4 Nursing Department, College of Applied Medical Science, King Faisal University, Hofuf, Saudi Arabia; 5 Department of Family and Community Health Nursing- Faculty of Nursing Science University of Khartoum, Khartoum, Sudan; 6 Nursing Science Department, College of Applied Medical Sciences, Prince Sattam Bin Abdulaziz University, Sudia Arabia; 7 College of Nursing Science, Department of Maternal and Child Health Nursing, Sekaka, Jouf University, Saudi Arabia

**Keywords:** conflict, nursing students, self-esteem

## Abstract

Armed conflict is a prevalent global issue that affects both the socioeconomic aspects of society and has profound psychological consequences for those directly involved. This cross-sectional comparative study explored the impact of armed conflict on the self-esteem of nursing students in Sudan and Saudi Arabia. In total, 308 nursing students provided data. The Rosenberg Self-Esteem Scale (RSES) was used to evaluate nursing students’ self-esteem. The findings demonstrated that nursing students who were exposed to armed conflict had lower self-esteem than those who were not. However, based on sex, marital status, and age, no statistically significant differences were observed in the nursing students’ self-esteem. The current findings indicate that nursing students exposed to armed conflict have low self-esteem. Interventions providing mental health support are essential for improving their self-esteem. Further research should explore other factors that could impact the self-esteem of nursing students.

## INTRODUCTION

Natural and manmade disasters are frequent worldwide [1], including numerous forms of armed conflict since the Second World War. Approximately 6% of the world’s population currently lives in locations affected by armed conflict [2], a type of manmade disaster linked to a high rate of morbidity and mortality, as well as unpredictable illnesses, injuries, and a high number of casualties [3]. The effects of armed conflict on civilians living in conflict and war zones have received considerable attention, suggesting that they often develop neuropsychiatric diseases such as depression, post-traumatic stress disorder (PTSD), suicidal ideation, and anxiety disorders [4]. In addition, the psychological impact of armed conflict can manifest as symptoms of dissociation, emotional instability, hostility, and cognitive impairment [5]. Research has also found that those exposed to violence during their lifetimes are more likely to develop various mental health problems [6].

While a large body of literature has examined the impact of armed conflict on psychological and mental health, the effects of armed conflict on self-esteem have received less attention, with research yielding contradictory results. However, its effect on nursing students’ self-esteem has not yet been examined. Studies by Patton *et al*. [7] and Mire *et al*. [8] found that those exposed to armed conflict have lower self-esteem than those who were not exposed. Conversely, Park *et al*. [9] found that individuals with more traumatic experiences had a higher level of self-esteem than those who had less traumatic experiences. However, this study was conducted after the participants had fled the conflict and arrived in the host country, which may have been associated with decreased depression, increased self-efficacy, and improved self-esteem.

Self-esteem refers to an individual’s positive or negative attitude toward himself or herself. According to Rosenberg, a person’s level of self-esteem conveyed their level of approval or disapproval, reporting that people with high self-esteem respected themselves, thought they were valuable, and were proud of their accomplishments. Conversely, those with low self-esteem frequently were found to lack confidence, be critical of themselves, and believe they were less competent and worthy than others [10]. Poor self-esteem is one of the strongest indicators of emotional and behavioral problems and has been linked to various youth problems, including antisocial behavior. Conversely, people with high self-esteem are typically happy and more likely to have close friendships [11].

Self-esteem is crucial for nurses because it improves their chances of success and is directly related to their communication skills [12]. Additionally, evidence indicates that people who experience an increase in their self-esteem are less likely to become anxious when faced with stressful events [13]. Nursing students work alongside nurses during disasters and war [14]. During clinical nursing practice, they are routinely subjected to highly stressful situations [15], including the need to address the dangers of conflict and its physical and psychological aftermath [16]. Research evidence indicates that nursing students in conflict-affected areas also report feelings of fear, helplessness, and inadequacy in their ability to provide care in challenging and dangerous environments [17]. This sense of powerlessness and lack of control over work conditions can negatively affect self-esteem. Therefore, building self-esteem may be valuable for nurses. If nurses can care for and help others, they might be expected to feel comfortable with themselves [18]; however, few studies have been conducted in Sudan that investigate the self-esteem of nursing students living in regions experiencing armed conflict. Therefore, the current study explored the self-esteem of nursing students exposed to armed conflict in Sudan compared to those in the Kingdom of Saudi Arabia. These findings could help build positive self-esteem among nursing students through a resilience program to improve their positive self-esteem and enhance their quality of life.

### Study hypothesis

H0: There would be no differences in self-esteem between nursing students who have been exposed to armed conflict and those who have not been exposed to armed conflict.

H1: There would be a difference in self-esteem between nursing students who have been exposed to armed conflict and those who have not been exposed to armed conflict.

## MATERIAL AND METHODS

### Study design and setting

This study utilized a cross-sectional comparative design to explore the impact of armed conflict on nursing students’ self-esteem. The study was conducted in two countries. The first study setting was Sudan in the Darfur and Kordofan regions, where the government universities are located. Both territories have experienced severe armed conflict, with increasing violence and an overall security situation that has remained tense and volatile since October 2021. According to the International Organization for Migration, over 83,000 people have been displaced because of the conflict in Darfur, and thousands have been displaced in Kordofan [19]. The study setting used for comparison was the Kingdom of Saudi Arabia, with governmental universities in the Riyadh region, which has not been affected by similar armed conflict. Data were simultaneously collected from August 2022 to March 2023.

### Participants

The sample was recruited from approximately 2,700 nursing students in the two settings (i.e., Sudan and Saudi Arabia). Approximately 1,500 students were from Sudanese government universities in Darfur and Kordofan, and approximately 1,200 were from Saudi government universities in Riyadh. The final number of participants included 308 nursing students (both women and men).

### Inclusion criteria

The inclusion criteria for the study required nursing students to attend a Sudanese government university in the Darfur or Kordofan regions or a Saudi government university in the Riyadh region. Participants needed to be enrolled in any of the last six semesters of their program and must either be residents of one of these regions or have relocated to these regions for their studies.

### Sample size and sampling method

A convenience sampling method was used in this study, where participants were selected based on their availability and accessibility to participate in the research. Data were collected manually and online via Google Forms based on participant accessibility. Participants from the Riyadh region were verbally informed by the researchers of the purpose, risks, and nature of the study before data collection and voluntarily agreed to participate. The questionnaire was mailed with a consent form for the participants from the Darfur and Kordofan regions, explaining that they could not respond until after reviewing and signing the consent form. A minimum sample size of 308 was calculated using the RaoSoft Digital Sample Size Calculator, and all participants met the inclusion criteria in this study.

### Definition of variables

#### Armed conflict

The term 'armed conflict’ is broad and can refer to a wide range of scenarios in which two groups of varying sizes engage in offensive or defensive armament use [20]. It is also defined as a type of manmade disaster associated with a high rate of morbidity and mortality, as well as unpredictable illnesses, injuries, and a staggering number of casualties [3].

#### Self-esteem

Self-esteem generally refers to a person’s evaluation of or attitude toward herself [21].

### Operational definitions

#### Exposure to armed conflict

In the context of this study, 'exposure to armed conflict’ was defined as the actual presence of nursing students in areas of armed conflict, experiencing the loss of civilian life, mass displacement, and violations of human rights and international humanitarian law.

#### No exposure to armed conflict

In this study, 'no exposure to armed conflict’ was defined as nursing students residing in secure zones and did not experience armed conflicts.

#### Armed conflict zones

In the context of this study, 'armed conflict zones’ referred to the Darfur and Kordofan regions, the two areas with the most severe conflicts in Sudan. An increase in violence, overall tense and volatile security situation, and mass displacement have occurred in Darfur and Kordofan, according to the International Organization for Migration [19].

### Measurement

Nursing students’ self-esteem was assessed using the previously developed and validated Rosenberg Self-Esteem Scale (RSES), a widely used self-report measure of self-esteem developed by Rosenberg in 1965 [10] that assesses an individual’s positive or negative attitudes toward oneself. The ten items on the RSES were scored on a 4-point Likert scale (1 = *strongly disagree*, 2 = *disagree*, 3 = *agree*, and 4 = *strongly agree*). The items were divided into two groups of five items. The first was linked with positively worded items related to feeling competent or self-aware, and the other five were linked with negatively worded items, using pejorative terms associated with sympathy with oneself [22]. The RSES has been extensively validated and used with various populations and settings, demonstrating good reliability and validity across different age groups and cultural backgrounds [23]. Schmitt and Allik evaluated the Rosenberg Self-Esteem Scale by translating it into 28 languages and administering it to 16,998 participants across 53 countries. They found the RSES reliable for assessing self-esteem across different languages and cultures [24]. In the standard interpretation of the RSES results, the global score, which is the overall score, is split into three potential levels of self-esteem after summing the individual scores for each item on the scale (scores ranging from 10 to 40 points). Scores of 10 to 25 indicated low self-esteem, 26 to 29 medium self-esteem, and 30 to 40 high self-esteem [25]. The scale’s internal consistency was good, as determined by a Cronbach’s alpha of 0.703. Additionally, we collected some demographic data, including gender, age, marital status, and exposure to armed conflict (with yes/no responses).

### Statistical analyses

Statistical analyses were performed using SPSS (version 25), which included descriptive and inferential statistics. The normality test of the data was performed using the Kolmogorov-Smirnov test, which showed the lack of normally distributed data. Therefore, a Mann–Whitney U test was used to analyze the data since the groups were independent and the data were not distributed normally. The significance level was set at *P* < 0.05.

## RESULTS

### Demographic characteristics

Of the 400 questionnaires distributed to the nursing students, 308 were returned, resulting in a response rate of 70%. These questionnaires were used for analysis. The response rate for the nursing students exposed to armed conflict was higher (211 participants) than that of nursing students who were not exposed to armed conflict (97 participants; 68.5%). Of all the participants, 207 (67.2%) were women, while 101 (32.8%) were men. They ranged in age from 18 to 37 years, with a mean age of 22.06 (SD = 2.87). More than two-thirds were exposed to armed conflict (68.5%, *n* = 211), and only 32 (10.4%) were married (Table 1).

**Table 1:** Demographic characteristics of the participants (n = 308)

Characteristics	*n* (%)
**Gender**	
Men	101 (32.8%)
Women	207 (67.2%)
**Marital status**	
Married	32 (10.4%)
Not married	276 (89.6%)
**Exposed to armed conflicts**	
Yes (Sudanese nursing students)	211 (68.5%)
No (Saudi nursing students)	97 (31.5%)
**Age**	
Minimum	18
Maximum	37
M ± SD^1^	22.06 ± 2.87

1 Mean ± standard deviation, frequency, and percentages (%)

### Self-esteem

In the standard interpretation of the RSES results, the global or overall score is split into three levels of self-esteem. The current results showed that nearly half of the respondents (48.7%) had high self-esteem. 26.0% had a medium level of self-esteem, and 25.3% had a low level of self-esteem (Table 2).

**Table 2:** Overall level of self-esteem of the participants based on their RES scores

Characteristics	*n* (%)
Low level of self-esteem	78 (25.3%)
Medium level of self-esteem	80 (26.0%)
High level of self-esteem	150 (48.7%)

### Comparison of mean self-esteem scores on the RSES by demographic characteristics and exposure to armed conflicts

We tested our hypothesis and examined the differences in self-esteem levels between nursing students exposed to armed conflict and those not exposed to armed conflict. We used the Mann–Whitney U test for two independent groups to compare mean self-esteem scores with demographic characteristics and exposure to armed conflict status. We found significant differences in the mean self-esteem scores of nursing students exposed to armed conflict compared to those who had not been exposed (Table 3). Nursing students who were not exposed to armed conflict had higher mean scores for self-esteem (mean rank = 177.23) than those exposed to armed conflict (mean rank = 144.05), with a significance level of *P* = 0.002. However, no statistically significant differences were observed in the mean self-esteem scores between genders (women: mean rank = 157.00; men: mean rank = 144.5; *P* = .730) or marital status (married: mean rank = 165.31; not married: mean rank =153.25; *P* = .467). The statistical significance was set at *P* < 0.05 (Table 4).

**Table 3:** Distribution of participant’s self-esteem responses on the RSE scale

Questions	Frequency (%)					
	Strongly Agree	Agree	Disagree	Strongly Disagree	Mean Score	Sth. Deviation
On the whole, I am satisfied with myself	153 (49.7%)	97 (31.5%)	48 (15.6%)	10 (3.2%)	3.28	.843
At times I think I am no good at all.	50 (16.2%)	98 (31.8%)	90 (29.2%)	70 (22.7%)	2.58	1.013
I feel that I have a number of good qualities.	169 (54.9%)	101 (32.8%)	24 (7.8%)	14 (4.5%)	3.38	.816
I am able to do things as well as most other people.	169 (54.9%)	90 (29.2%)	35 (11.4%)	14 (4.5%)	3.34	.853
I feel I do not have much to be proud of.	45 (14.6%)	80 (16.6%)	63 (20.5%)	120 (39.0%)	2.84	1.101
I certainly feel useless at times.	43 (14.0%)	93 (30.2%)	80 (26.0%)	92 (29.9%)	2.72	1.041
I feel that I’m a person of worth.	111 (36.0)	91 (29.5%)	61 (19.8%)	45 (14.6%)	2.87	1.063
I wish I could have more respect for myself.	144 (46.8)	71 (23.1%)	40 (13.0%)	53 (17.2%)	2.01	1.136
All in all, I am inclined to think that I am a failure.	42 (13.6%)	61 (19.8%)	84 (27.3%)	121 (39.3%)	2.92	1.065
I take a positive attitude toward myself.	172 (55.8%)	86 (27.9%)	31 (10.1%)	19 (6.2%)	3.33	.893

**Table 4:** Comparison of mean self-esteem scores on the RSE scale by demographic characteristics and exposure to armed conflicts

Categories	Group	*n* (%)	Mean rank	Sum of ranks	Mann–Whitney U	*P* value
Exposed to armed conflict	Yes	211 (68.5%)	144.05	30394.50	8028.500	.002
	No	97 (31.5%)	177.23	17191.50		
Gender	Men	101 (32.8%)	157.00	15857.50	10200.500	.730
	Women	207 (67.2%)	153.28	31728.50		
Marital status	Married	32 (10.4%)	165.31	5290.00	4070.000	.467
	Not married	276 (89.6%)	153.25	42296.00		

## DISCUSSION

This study’s findings provide valuable insights regarding the impact of armed conflict on nursing students’ self-esteem. To the best of our knowledge, this is the first study that examines the differences in the self-esteem of nursing students who were exposed to armed conflict and those who were not by comparing students from Sudan (who had exposure to armed conflict) and Saudi Arabia (who have not been exposed to armed conflict). Our results indicated that nursing students who were exposed to armed conflicts had lower self-esteem levels than those who were not exposed to armed conflicts, consistent with previous research indicating that students exposed to violence had low self-esteem [7,8]. Research has demonstrated that exposure to stress, crises, political conflicts, and violence reduces self-esteem [26,27]. Consequently, individuals exposed to armed conflict may experience low self-esteem due to the associated stress. This finding is supported by a substantial body of scientific research highlighting the negative mental and psychological consequences of armed conflict [5,28–32]. The fact that nursing students exposed to armed conflict have lower self-esteem is concerning, as self-esteem plays a crucial role in their mental and emotional well-being. This finding underscores the need for interventions and support systems to help individuals who have experienced armed conflict improve their self-esteem and overall mental health. The discrepancy in self-esteem levels among nursing students based on their exposure to armed conflict raises questions about the different coping mechanisms and resilience factors that individuals may develop in response to traumatic events. Conversely, a study by Park *et al*. discovered that an increased number of traumatic experiences was related to greater levels of self-esteem [9]. This finding might be explained by the study’s context, which involved people fleeing regions of armed conflict to safe areas. Thus, they may view themselves as having a higher status than the negative life events experienced before they arrived in the host country, which may be associated with lower depression, increased perceived self-efficacy, and improved self-esteem [33].

We tested our hypothesis by comparing the overall mean self-esteem scores with demographic characteristics and exposure to armed conflict status to determine differences in self-esteem. We found that nursing students who were not exposed to armed conflict had higher mean self-esteem scores (30.78 ± 5.3) than those who were exposed to armed conflict (28.57 ± 4.9). Our results also showed that age had no significant effect on adult self-esteem, aligning with several cross-sectional, longitudinal, and sequential cohort studies that demonstrated age-related increases in self-esteem in both men and women from late adolescence to middle adulthood [34]. Self-esteem tends to stabilize before declining in old age [35], which could explain the current findings since the respondents were adults. Another interesting finding of this study was the lack of significant differences in overall self-esteem between men and women, consistent with the findings of Sinclair *et al*. [36]. In the current study, no significant differences in overall self-esteem were found by marital status, which is inconsistent with a study in the United States that reported that married mothers had higher self-esteem than unmarried mothers [37]. The lack of significant differences in overall self-esteem based on age, gender, and marital status in this study is interesting and requires further exploration. The absence of these group differences may suggest that factors such as exposure to armed conflict have a stronger impact on self-esteem than traditional demographic variables. Future research should examine other factors that could influence self-esteem, such as social support, coping strategies, and previous exposure to trauma.

Our study did not consider the effect of educational level on self-esteem. However, several studies have identified a positive correlation between education and self-esteem, indicating that individuals with higher educational levels tend to have higher self-esteem [38]. Since all the participants in our study were college students, this finding supports the hypothesis that armed conflict affects self-esteem. Evidence is conflicting regarding the relationship between educational levels and self-esteem since some research suggests that individuals with lower levels of education have higher self-esteem [39].

### Limitations

Some limitations of this study must be acknowledged. First, causation could not be determined as this was a correlational study. It is still unclear whether nursing students who have been exposed to armed conflict have lower levels of self-esteem before their exposure to armed conflict or are generally more likely to have low self-esteem compared to those who have not been exposed to armed conflict. Additionally, the sample was limited to nursing students, so it may be useful to expand the sample size to include all nurses in future studies.

### Recommendations

These findings suggest several recommendations for universities and academic institutions regarding the implementation of comprehensive psychosocial and preventive interventions that are focused on improving self-esteem and well-being and mitigating the negative effects of armed conflict on students. Additionally, further research should explore various factors that might influence nursing students’ self-esteem.

### Study implications

This study emphasizes the impact of armed conflict on nursing students’ self-esteem, which has not been adequately explored in previous studies. By specifically examining this relationship, the study provides valuable insights regarding how the stress and trauma of armed conflict can affect nursing students’ self-perception and confidence. Additionally, by comparing the self-esteem of nursing students in conflict-affected areas to those in non-conflict areas, this study provides a nuanced understanding of the specific challenges faced by nursing students in conflict zones. Overall, this study contributes to the existing literature on the psychological impact of armed conflict on healthcare professionals and provides strategies to support the mental health and well-being of nursing students in conflict-affected areas.

## CONCLUSION

This study revealed that nursing students exposed to armed conflict (i.e., Sudanese nursing students) had lower self-esteem than nursing students who were not exposed to armed conflict (i.e., Saudi nursing students). The findings demonstrated that nursing students can be greatly affected by their exposure to armed conflict and that these experiences are a substantial independent risk factor for lowering their level of self-esteem.

## Data Availability

Further data are available from the corresponding author upon reasonable request.

## Personal thanks

The authors thank the participants who voluntarily participated in this study and the administrators who helped conduct this research.
